# Gas-Phase Ions from Neutral Microdroplets

**DOI:** 10.1021/acscentsci.5c02140

**Published:** 2025-11-24

**Authors:** Ochir Ochirov, Pawel L. Urban

**Affiliations:** Department of Chemistry, 34881 National Tsing Hua University, 101, Section 2, Kuang-Fu Rd., Hsinchu, 300044, Taiwan

## Abstract

Contact electrification and mechanical breakup can charge neutral water droplets, providing a pathway for gas-phase ion formation.

## Graphical abstract

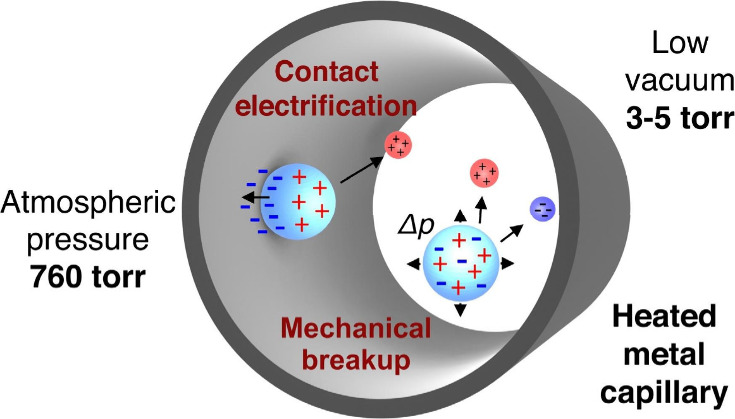

In this issue of *ACS Central Science*, Williams and co-workers^1^ report that neutral water droplets are accelerated in atmospheric sampling mass spectrometers, gaining high kinetic energies, and become activated by contact electrification at the ion transfer capillary surface. These events lead to the production of highly charged positive droplets. The heated ion transfer capillary—involved in this process—is a common part of highly popular mass spectrometers equipped with an atmospheric pressure interface. In this study, droplets were generated in three ways: (i) by electrospray ionization (ESI), (ii) by a mechanical vibrating mesh nebulizer, and (iii) by condensation of water molecules into *neutral* microdroplets in ambient air above an open liquid nitrogen dewar. To investigate the droplet properties, the authors used a custom-built charge detection instrument. Interestingly, while the majority of highly charged droplets originating from (iii) were positively charged, a small fraction of them were negatively charged (Figure 1).

**Figure 1:**
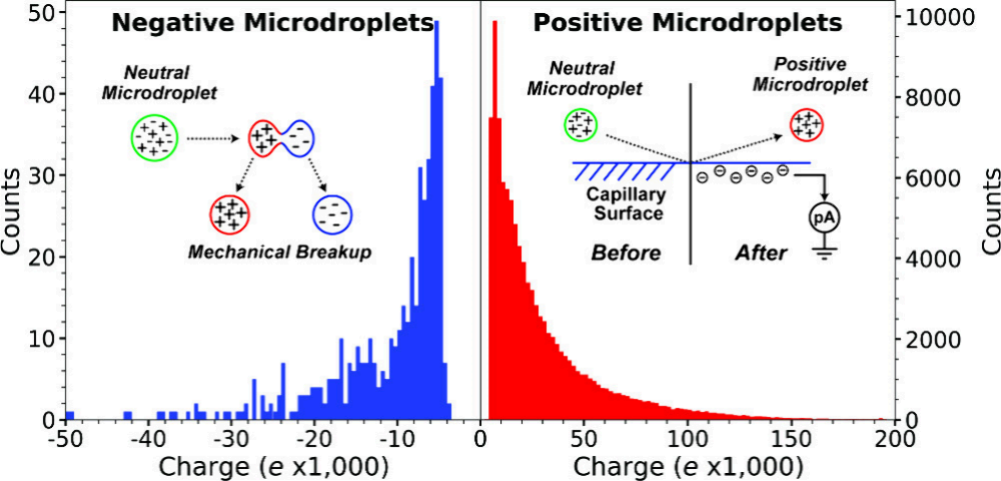
Population of droplets originating from neutral droplets generated by condensation with liquid nitrogen as a function of individual droplet charge. Negatively and positively charged droplet data are shown in blue and red, respectively. Reproduced from ref 1. Available under a CC-BY 4.0 license. Copyright 2025 Matthew S. McPartlan, Casey J. Chen, Conner C. Harper, Zachary M. Miller, Julian Robles, Veena S. Avadhani, Randall E. Pedder, Luke J. Metzler, and Evan R. Williams.

Following its over 100-year-long history, mass spectrometry (MS) has been elevated to become one of the most prominent analytical techniques. This success is attributed to the development of ESI and numerous ambient ionization techniques.^2^ In ESI, liquid samples are passed through a capillary while applying high voltage and dispersed to the atmospheric pressure. Auxiliary (nebulizing, drying) gases are often used to assist dispersion and evaporation processes. Some of the resulting highly charged micrometer-scale droplets undergo a fission process, which was theorized by Lord Rayleigh in the late 19th century.^3^ Gas-phase ions are eventually formed from the charged nanometer-scale droplets via the accepted mechanisms: ion evaporation model, charged residue model, or chain ejection model.^4^ In the past several years, various experiments were carried out to verify the possibility of generating ions in similar setups but without applying voltage to the sample emitter or even without intentional spraying. To date, ion formation from neutral droplets and the influence of the atmospheric sampling interface (e.g., the inlet capillary) have not been understood completely.

In their report, Williams and co-workers cast more light on the formation of highly charged species of both polarities from neutral droplets, proposing two comprehensive mechanisms.^1^ The formation of positive droplets is predominantly due to the contact electrification, when a steel capillary inner surface seizes electrons and anions from neutral droplets, providing charges up to +200,000 e. The formation of fewer negative droplets is explained by bipolar mechanical breakup of neutral droplets. It should be noted that the latter mechanism was previously relied upon to develop an ionization technique for MS, which supports the notion that elevated temperatures enhance the internal pressure of a droplet, thereby promoting breakup events.^5^ Moreover, in our view, the discussed droplet charging mechanism complements the statistical charging theory,^6^ which was previously used to explain ion formation in thermospray ionization^7^ and some newer ionization techniques. In fact, other ionization techniques were reported, in which liquid droplets are dispersed without applying voltage to the sample emitter (e.g., ref^8^). In their discussion, Williams and colleagues call into doubt the necessity of a strong intrinsic droplet electric field in both the spontaneous formation of charged species from neutral droplets and unusual microdroplet chemistry.^1^ Their study, in turn, points to the role of a solid–liquid interface in the ion source for ion generation. Therefore, it aligns well with the findings of McEwen, Trimpin, and co-workers who previously devised “inlet ionization”^9^ – a technique in which a liquid sample directly interacts with the surface of the ion transfer capillary.

The formation of positive droplets is predominantly due to the contact electrification, when a steel capillary inner surface seizes electrons and anions from neutral droplets, providing charges up to +200,000 e. The formation of fewer negative droplets is explained by bipolar mechanical breakup of neutral droplets.

A notable feature of this and some other studies conducted by the same group is the use of the custom image charge detector within the turbopumped region of a mass spectrometer. This method permits direct measurements of the charge and velocity of highly charged droplets, enabling estimation of their diameters and masses. These quantitative findings lead to a deeper understanding of the mechanisms underlying microdroplet behavior, which is invaluable for various chemistry fields, including spray- and electrospray-based ionization MS and microdroplet chemistry.

Following the authors’ discussion, one can further speculate whether the contact electrification and mechanical breakup of droplets may also contribute to ion formation in conventional ESI. These processes are probably responsible for improved sensitivity of conventional ESI because many electrospray microdroplets are not sufficiently charged to undergo Rayleigh fissions or are too large to be completely desolvated through evaporation. Thus, the study provides a more complete picture of the ion formation process under standard ESI-MS conditions. Nonetheless, it should be noted that, in the Williams’ study, all ion optics in the main experiments were grounded.^1^ This eliminates the influence of electric fields as a potential confounding factor while explaining droplet movement and charging. In commercial mass spectrometers, ion optic elements are supplied with voltages. This difference constrains extrapolating the findings to common MS scenarios. Another limitation concerns the lower and upper bounds of droplet size and charge in the charge detection instrument.

Using the developed cryogenic ionization MS, mass spectra were obtained for selected (semi)volatile organic compounds (VOCs).^1^ This process resembles secondary electrospray ionization MS, which may involve either gaseous ion–molecule reactions or vapor–droplet interactions (Figure 2).^10^ In principle, it is imaginable that the VOC ions are produced following initial dissolution of the analytes in the water droplets or due to proton transfer involving dry gaseous species. The high abundances of both doubly and singly charged ions in the Williams’ work indicate that both proposed mechanisms of droplet charging are plausible.

**Figure 2:**
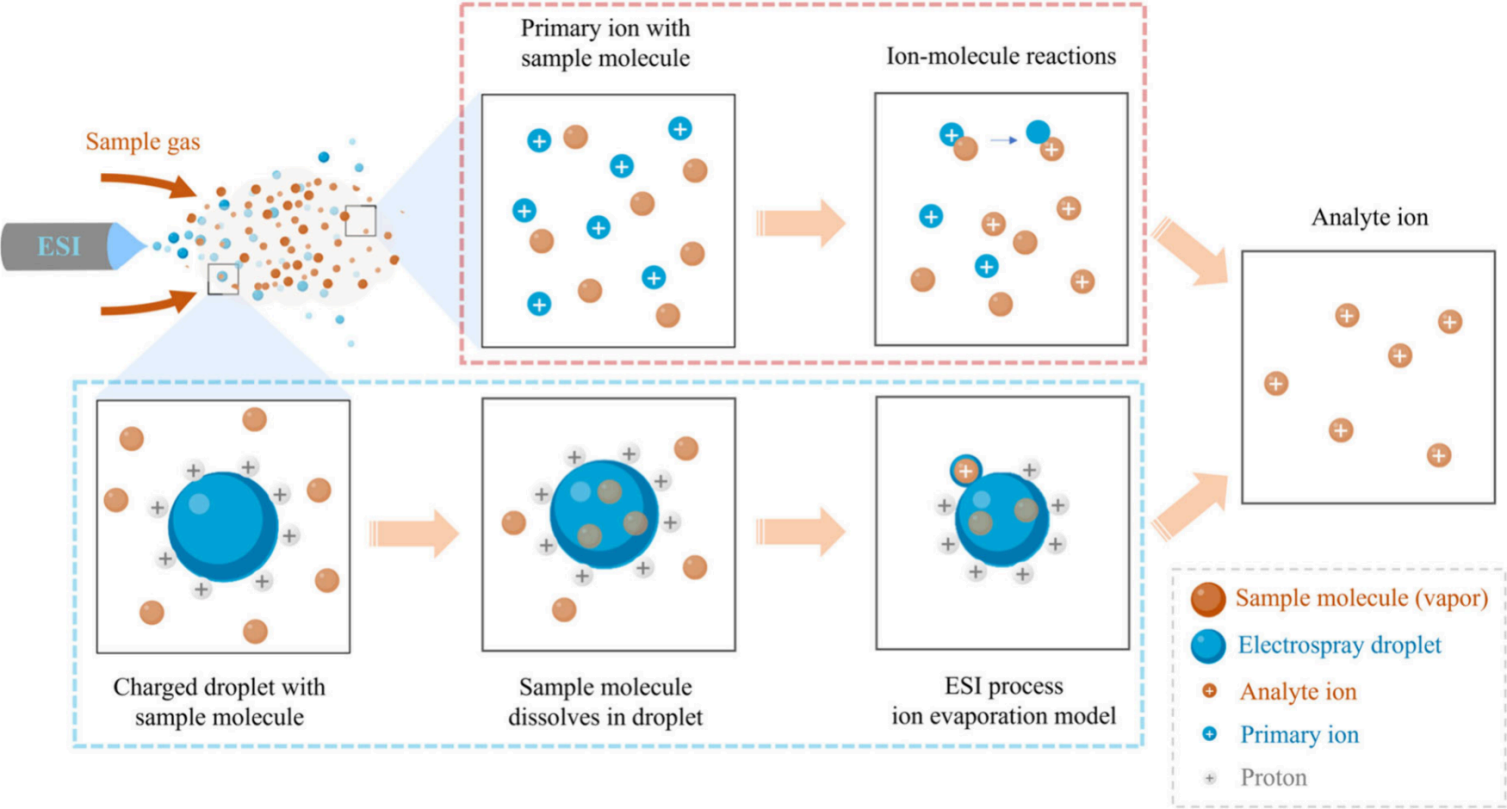
Two possible ionization mechanisms in a secondary electrospray ionization MS source: gaseous ion–molecule reaction (within the red dashed box); vapor–droplet interaction (within the blue dashed box). Reproduced with permission from ref 10. Copyright 2025 John Wiley & Sons.

The processes observed by the Williams group may be significant for ion formation in previously disclosed ion sources, including ESI and ambient ion sources.

In conclusion, while the practicality of the approach as a new common-use ionization technique is arguably limited, the study provides unique mechanistic insights. The processes observed by the Williams group may be significant for ion formation in previously disclosed ion sources, including ESI and ambient ion sources. They also contribute to the understanding of “microdroplet chemistry” and the use of microdroplets as vessels for chemical reactions. It is possible that undercharged droplets—produced by electrosprays—give rise to gas-phase ions following the pathway proposed for the cryogenic ionization MS. This additional mechanism may enhance ion signals and the sensitivity of common ESI-MS methods. In the long run, the reported insights can be taken into account by the developers of commercial mass spectrometers, thus indirectly contributing to better instruments.
